# High-dose interleukin-2 (HD IL-2) for advanced melanoma: a single center experience from the University of Pittsburgh Cancer Institute

**DOI:** 10.1186/s40425-017-0279-5

**Published:** 2017-09-19

**Authors:** Diwakar Davar, Fei Ding, Melissa Saul, Cindy Sander, Ahmad A. Tarhini, John M. Kirkwood, Hussein A. Tawbi

**Affiliations:** 1 University of Pittsburgh Cancer Institute and University of Pittsburgh Medical Center, Pittsburgh, PA USA; 2 Department of Biostatistics, University of Pittsburgh and University of Pittsburgh Medical Center, Pittsburgh, PA USA; 3 Clinical Research Informatics, University of Pittsburgh and University of Pittsburgh Medical Center, Pittsburgh, PA USA; 4 Clinical and Translational Science, University of Pittsburgh Cancer Institute and University of Pittsburgh Medical Center, Pittsburgh, PA USA; 5 Dermatology, and Clinical and Translational Science, University of Pittsburgh Cancer Institute and University of Pittsburgh Medical Center, Pittsburgh, PA USA; 6 Department of Melanoma Medical Oncology, Division of Cancer Medicine, The University of Texas MD Anderson Cancer Center, Houston, TX USA; 7 Division of Hematology-Oncology, University of Pittsburgh Medical Center, 5117 Centre Avenue, Pittsburgh, PA 15232 USA

**Keywords:** Melanoma, Metastatic, Interleukin-2, HD IL-2, Immunotherapy, CTLA-4, PD-1, BRAF, MEK

## Abstract

**Background:**

Durable remissions are observed in a fraction of metastatic melanoma patients treated with high-dose interleukin-2 (HD IL-2). Early studies reported overall (OR) and complete response (CR) rates of 16% and 8% respectively. Toxicity limited use to specialized centers with standardized protocols. We report on 243 patients treated at the University of Pittsburgh in a non-intensive care unit (ICU) oncology specialty setting.

**Methods:**

Clinical and radiological data were collected on 243 patients treated between 1992 and 2015. Each HD IL-2 cycle was given over 5 days, cycles were repeated after 9 days and courses (2 cycles) were repeated every 6–9 weeks in patients with stable or responding disease, for up to 3 courses total. Influence of baseline characteristics on outcomes was assessed using Kaplan-Meier estimates and Cox proportional hazards analysis.

**Results:**

Two hundred forty-three patients received 692 cycles (5270 doses) between 1992 and 2015. Two hundred thirty-seven patients were evaluable for response: OR rate 18.1% with CR rate 8.0%. Median overall survival (OS) 9.6 months in the entire cohort but 64.9 months in responders. Median number of cycles delivered was 2,and median number of doses per cycle was 8. Toxicity was consistent with prior reports. HD IL-2 required ICU transfers in 11 patients and 1 death was attributed to HD IL-2. Pre-treatment lactate dehydrogenase (LDH) levels correlated significantly with progression-free survival [1-2× upper limit normal (ULN) HR 1.95; >2× ULN HR 2.32] and overall survival (1-2× ULN HR 1.67; >2× ULN 2.49). Response to HD IL-2 and site of metastatic disease also correlated significantly with progression-free and overall survival.

**Conclusions:**

In this large series of patients spanning more than two decades, OR/CR rates with HD IL-2 were 18.1%/8.0% respectively. Toxicity data was consistent with prior reports. Pre-treatment LDH values and site(s) of metastatic disease may be useful markers to select patients at greater likelihood of benefit to HD IL-2 therapy.

**Electronic supplementary material:**

The online version of this article (10.1186/s40425-017-0279-5) contains supplementary material, which is available to authorized users.

## Background

Interleukin-2 is a T-cell growth factor with well characterized effects on growth and expansion of T-cell subsets particularly CD8+ T-cells and documented anti-tumor efficacy in advanced renal cancer and melanoma [1, 2]. Advanced melanoma is a disease previously considered incurable with limited treatment options and a median overall survival (OS) historically estimated at 8 to 10 months. Hitherto, high-dose interleukin-2 (HD IL-2) was the only approved immunotherapy for stage IV melanoma - based on durable long-term survival observed in a fraction of patients initially reported in a phase II study in 1994, further updated in a meta-analysis of phase II trials published in 1999 [3, 4]. In the latter study, authors reported 17 complete (6.3%) and 26 partial (9.6%) responses for an objective response (OR) rate of 15.9% in 270 treated patients. 12 of 43 initial responders (27.9%, 10 of whom were complete responders) remained progression-free at the time of reporting, with a plateau in OS after 36 months. Commonest toxicity associated with HD IL-2 is hypotension, secondary to underlying capillary leak, reduced peripheral vascular resistance and increased cardiac output similar to a systemic inflammatory response syndrome (SIRS) syndrome that reflects the mechanism of action of IL-2. This, together with the routine use of vasopressors to maximize dose intensity contributed to a high incidence of grade 3/4 toxicities and mandated therapy in a highly regulated setting, commonly an intensive care unit (ICU).

The preceding decade has witnessed unprecedented advancements in our understanding of both the molecular drivers of melanoma tumorigenesis and mechanisms by which tumors hijack negative regulatory checkpoints to circumvent anti-tumor immunity; leading to the development of targeted inhibitors of mitogen activated protein kinase (MAPK) signal transduction pathway and negative regulatory immune checkpoints. Seven new agents including BRAF inhibitors (vemurafenib and dabrafenib), MEK inhibitor (trametinib and cobimetinib), anti-cytotoxic T-lymphocyte antigen 4 (CTLA-4) inhibitor ipilimumab, programmed death 1 (PD-1) inhibitors (nivolumab and pembrolizumab) and talimogene laherparepvec have demonstrated improved survival and are approved singly and in combination for the management of advanced melanoma [5–14]. Following the approval of BRAF/MEK inhibitors and PD-1/CTLA-4 blocking antibodies starting in 2011, the use of HD IL-2 in the frontline therapy of melanoma gradually declined.

Little is known about how best to sequence these agents though limited reports suggest that patients who progress past HD IL-2 can be salvaged with CTLA-4 and/or PD-1 inhibitors though these numbers are small. At the University of Pittsburgh Cancer Institute, we implemented a protocol for the administration of HD IL-2 in a non-ICU oncology specialty setting in 1994. To provide estimates of the toxicity and efficacy of this method of administration, we conducted a retrospective analysis of response, survival and toxicity data of 243 advanced melanoma patients treated with HD IL-2 between 1992 and 2015.

## Methods

### Patient selection

Approval was obtained from the University of Pittsburgh Cancer Institute (UPCI) Institutional Review Board (IRB) for a retrospective analysis of patients with advanced melanoma who received treatment with HD IL-2 (IRB number PRO13050140). Patients treated between March 1992 and June 2015 were included in this analysis. Patients received HD IL-2 either as a standard-of-care (SOC) therapy (193 patients) or on one of two studies: UPCI 03–137 (HD IL-2 with sequential temozolomide - 30 patients) [15, 16] and UPCI 10–095/NCI 8628 (HD IL-2 with or without ziv-aflibercept - 60 patients). Of the 60 patients treated in UPCI 10–095/NCI 8628, 20 patients treated with HD IL-2 alone were included, while the 40 patients treated with HD IL-2/ziv-aflibercept were excluded from this analysis. Detailed reports of these studies have been published previously.

### Drug administration

In all instances (SOC and UPCI studies 03–137/10–095), HD IL-2 was dosed at 600,000 IU/kg administered by intravenous infusion over 15-min every 8 h for up to 14 consecutive doses over 5 days. Orders detailing maintenance fluids, prophylactic antibiotics and laboratory monitoring were developed and entered on admission for all patients (available on request). Published guidelines were used to guide administration or withholding of each dose [17]. Use of low-dose dopamine (up to 5 μg/kg/min) was permitted to maintain urine output though vasopressors and/or ventilatory support were not used to maximize dose intensity. Therapy was held for commonly accepted relative and absolute dose-limiting toxicities (DLT); and terminated at patients’ request, if ≥3 doses were held for relative DLT, or upon the development of 1 or more absolute DLTs [17]. Primary DLT(s) leading to cessation of therapy were recorded and tabulated.

### Patient and clinical characteristics and dosing details

In this retrospective analysis, descriptive statistics (including medians and range) were used to summarize demographic, laboratory and histopathologic variables. Primary site was classified as cutaneous, mucosal, uveal or unknown when patients presented with metastatic disease in the absence of a clear primary lesion. Extent of metastatic disease was staged according to AJCC 2009 staging system for M1a (cutaneous, sub-cutaneous and nodal metastases) and M1b (pulmonary metastases) but we further divided M1c (visceral metastases) based on the presence of absence of central nervous system (CNS) disease (M1c non-CNS and M1c CNS) [18]. Pre-treatment lactate dehydrogenase (LDH) values were abstracted and trichotomized into normal, 1-2× upper limit normal (ULN) and >2× ULN. Details regarding number and nature of pre-HD IL-2 therapies and post-HD IL-2 therapies were obtained and tabulated. Pharmacy records were reviewed for dosing details and reason(s) for discontinuation of therapy.

### Toxicity evaluation, response assessment, determination of survival/progression and statistical analyses

Toxicity assessments were graded according to National Cancer Institute Common Terminology Criteria for Adverse Events version 3.0. DLT(s) leading to cessation of therapy were abstracted from the electronic health record. Disease response was determined using the Response Evaluation Criteria in Solid Tumors (RECIST v1.1) guidelines [19]. Progression-free survival (PFS) was defined as time from start of HD IL-2 therapy to radiographic progression, clinical progression, or death, whichever occurred first. Where possible, progression was characterized as systemic-only, CNS-only or systemic and CNS. OS was defined as time from HD IL-2 initiation to death. The influence of baseline patient demographic and tumor characteristics on treatment outcomes was assessed using Cox proportional hazards analysis. Kaplan-Meier methods were used to generate estimates for PFS and OS along with corresponding 95% confidence intervals (CI). Data cutoff was defined as at 1/30/2017. All statistical analyses were performed post hoc and not adjusted for multiple testing.

## Results

### Patients and clinical characteristics

Between March 1992 and June 2015, data from 243 patients with American Joint Committee on Cancer (AJCC) stage IV melanoma who had received at least 1 cycle of HD IL-2 were aggregated. Baseline characteristics of all 243 patients are detailed in Table 1. 55% of patients were male with a median age of 48 years. 77% (188/243) of treated patients had cutaneous melanoma while a minority of patients had mucosal (6%, 14/243), uveal (7%, 16/243) or unknown primaries (10%, 24/243). 18% of patients had metastatic disease limited to skin, subcutaneous tissue and/or lymph nodes (M1a) while 24% of patients had pulmonary metastases (M1b) and 18% had treated central nervous system (CNS) metastases. The remaining 40% had non-lung visceral metastases. At the time of data cutoff, median duration of follow up was 9.4 months (range 0.2 to 273 months).

**Table 1:** Baseline characteristics

	*N* = 243
Median Age (range) – yr.	48 (14–77)
Sex – no. (%)	
Male	133 (55)
Female	110 (45)
Primary Site – no. (%)	
Cutaneous	188 (77)
H&N	34 (14)
LE	38 (16)
UE	29 (12)
Breast	1 (<1)
Trunk – anterior/posterior	74 (30)
Unknown	12 (5)
Mucosal	14 (6)
GI - anorectal	3 (1)
Aerodigestive	3 (1)
Urethral	1 (<1)
Vulva	7 (3)
Unknown	24 (10)
Uveal (choroid)	16 (7)
Other (orbit)	1 (<1)
Metastatic status prior to HD IL-2 – no. (%)	
Skin, subcutaneous, LN (M1a)	43 (18)
Lung (M1b)	58 (24)
Non-lung visceral (M1c non-CNS)	99 (41)
CNS (M1c CNS)	43 (18)
LDH – no. (%)	
Normal	106 (44)
Abnormal	137 (56)
1xULN-2xULN	74 (30)
> 2xULN	63 (26)
Protocol – no. (%)	
Standard of care (SOC)	193 (79)
HD IL-2/temozolomide	30 (12)
HD IL-2+/− ziv-aflibercept (HD IL-2 alone)	20 (8)
Line of therapy – no. (%)	
1st line	114 (47)
2nd line	83 (34)
3rd line or subsequent line	46 (19)
Pre-HD IL-2 therapies* – no. (%)	
CTLA-4 inhibitor	20 (8)
PD-1 inhibitor	2 (1)
Other immunotherapies (including biochemotherapy)	73 (30)
BRAFi/MEKi target therapy	3 (1)
Other targeted therapy	13 (5)
Chemotherapy	54 (22)
Post-progression therapies* – no. (%)	
CTLA-4 inhibitor	29 (12)
PD-1 inhibitor	11 (5)
Other immunotherapies (including biochemotherapy)	29 (12)
BRAFi/MEKi target therapy	12 (5)
Other targeted therapy	5 (2)
Chemotherapy	43 (18)

*These categories are not mutually exclusive or exhaustive

### Toxicity profile

Two hundred forty-three patients received 5270 doses of HD IL-2 over 692 cycles in total. The number of patients who continued to receive HD IL-2 diminished with successive cycles – either for progression or toxicity. Per patient, median number of cycles received was 2, median number of total doses received was 17 and median number of doses per cycle received was 8. HD IL-2 administration resulted in 11 admissions to intensive care unit (ICU) (12/692 cycles, 1.6% incidence) most commonly for hypotension non-responsive to fluid resuscitation requiring vasopressor administration and hypoxemia secondary to pulmonary capillary leak syndrome. 1 death was attributed to HD IL-2 (0.4% mortality). Incidence of relative and/or absolute DLTs that led to termination of HD IL-2 therapy were obtained and are tabulated in Additional file 1: Table S1. Toxicity profile was consistent with prior reports of this agent. Across all cycles, oliguria (14%–58%), hypotension (14%–39%) and tachycardia (10%–21%) accounted for the majority of relative/absolute DLTs.

*Response Analysis.*

Of 243 treated patients, 237 patients were evaluable for response (summarized in Table 2). Six patients were not evaluable for response. Complete responses (CR) was observed in 19 (8%) while partial responses (PR) was observed in 24 (10%) for an overall response rate (ORR) of 18% (95% CI 13%–24%). Additionally, 54 patients (23%) had stable disease for a disease control rate (DCR) of 41% (95% CI 35%–47%). Response did not differ significantly by site of primary tumor though the number of non-cutaneous uveal/mucosal primaries was low in this series (see Additional file 1: Table S2). Of the 19 patients who had a CR to HD IL-2; 3 patients subsequently relapsed, 1 of whom passed away after developing CNS disease. A 2nd patient developed an isolated biopsy-proven subcutaneous recurrence which was resected and irradiated and remains disease-free; while the 3rd patient achieved a durable remission with fourth-line PD-1 inhibitor therapy with pembrolizumab following progression on vemurafenib/PI3K inhibitor PX-866 and CTLA-4 inhibitor ipilimumab following progression on initial HD IL-2.

**Table 2:** Tumor response to HD IL-2

	Entire Cohort (*N* = 237)	Cutaneous (*N* = 182)	Mucosal (*N* = 14)	Uveal (*N* = 16)	Other (*N* = 1)	Unknown (*N* = 24)
Best Response – no. (%)						
CR	19 (8)	17 (9)	1 (7)	0 (0)	0 (0)	1 (4)
PR	24 (10)	18 (10)	2 (14)	1 (6)	0 (0)	3 (13)
SD	54 (23)	37 (20)	4 (29)	5 (31)	0 (0)	8 (33)
PD	140 (59)	110 (60)	7 (50)	10 (63)	1 (100)	12 (50)
No. of patients with CR or PR	43	35	3	1	0	4
Percentage (95% CI)	18 (13–24)	19 (14–26)	21 (5–51)	6 (0–30)	0 (−)	17 (5–37)
No. of patients with CR, PR, or SD	97	72	7	6	0	12
Percentage (95% CI)	41 (35–47)	40 (32–47)	50 (23–77)	38 (15–65)	0 (−)	50 (29–71)

We observed that the median number of doses administered differed by response category. Unsurprisingly, patients with complete/partial responses received statistically significant greater doses (median = 33), than patients with stable disease or non-responders (median = 16) (*p* < 0.0001).

*BRAF and NRAS* mutation status were known on 51 and 37 patients, respectively. ORR was 31% (95% CI 15%–51%) in *BRAF* mutant compared to 14% (3%–35%) in *BRAF* wild type patients. Although this difference was not statistically significant, it is consistent with prior data suggesting greater response rates in *BRAF/NRAS* mutant patients compared to wild type patients [20]. Given the small number of *NRAS* mutant patients, differential response statistics between *NRAS* mutant and wild type patients cannot be interpreted. Although the response rate was greater in the 1st line (23%) than in the 2nd or subsequent line (14%) – this difference was not statistically significant.

### PFS and OS analyses

The primary analysis of 243 patients revealed a median OS of 9.6 months (95% CI, 7.4 to 11.2 months) in the entire cohort but 64.9 months (95% CI, 28.2-infinity) in responders. 1-, 2- and 3- year survival rates were 41%, 20% and 14% respectively. Median PFS was 2.8 months (95% CI 2.2–3.5) after excluding 6 patients deemed unevaluable for progression. Median follow-up time was approximately 9.4 months (range 0.2 to 273 months) at the time of data-cutoff. Primary analysis included 19 complete responders with a median follow-up time of 88.9 months (range 3.6 to 273 months). Of these, 3 patients progressed, 2 of whom were subsequently salvaged as above. Two patients with CRs passed away - though only 1 death was related to melanoma recurrence.

**Fig. 1:**
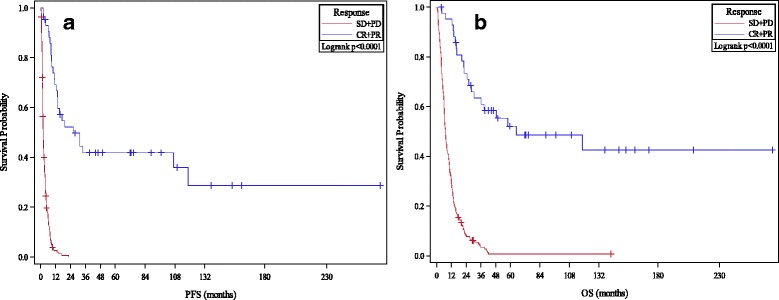
PFS and OS Analyses By Response to HD IL-2 Therapy. **a** and **b** Kaplan-Meier plots of progression free survival (**a**) and overall survival (**b**) after HD IL-2 therapy are compared by response to therapy (CR/PR vs. SD/PD). All *p*-values significant

In comparing 1−/2−/3- year response rates for responders and non-responders, we considered two categories of responders: first excluding patients with stable disease (CR/PR only) and second including patients with stable disease (CR/PR/SD). 1−/2−/3- year OS rates for CR/PR patients were 95%/73%/63%; while 1−/2−/3- year PFS rates for CR/PR patients were 69%/52%/42% respectively. When patients with stable disease were included as responders, 1−/2−/3- year OS rates for CR/PR/SD patients were 71%/41%/31%; while 1−/2−/3- year PFS rates for CR/PR/SD patients were 35%/23%/19% respectively. Kaplan-Meier curves for PFS and OS by response are presented in Fig. 1. Potentially prognostic factors are delineated in detail in Additional file 1: Tables S3 and S4.

**Fig. 2:**
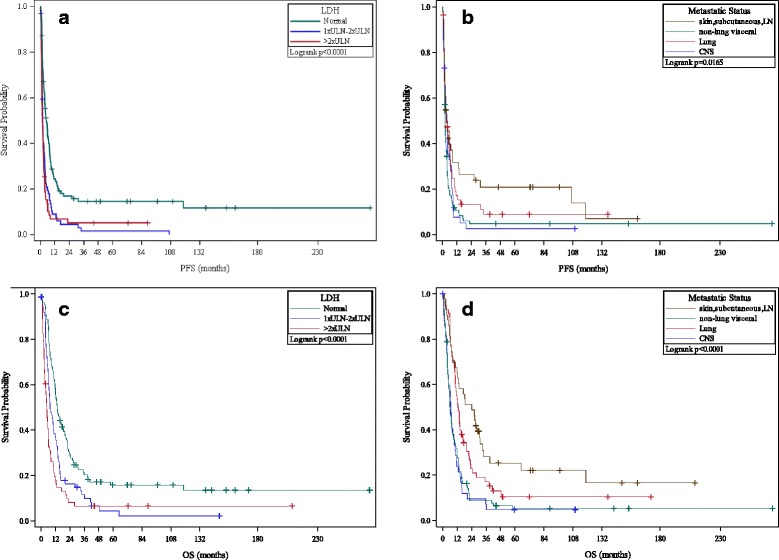
OS/PFS Analyses By LDH and Pre-Treatment Disease Burden. **a** and **b** Kaplan-Meier plots of progression free survival after HD IL-2 therapy in melanoma patients compared by extent of pre-treatment LDH levels (**a**) and site of metastatic disease (**b**). **c** and **d** Kaplan-Meier plots of overall survival after HD IL-2 therapy in patients compared by pre-treatment LDH levels (**c**) and site of metastatic disease (**d**). All *p*-values significant and unadjusted for multiple comparisons

Both absolute LDH elevation and the extent of LDH elevation were correlated with worse PFS compared to patients with normal LDH: 1-2× ULN (HR 1.95, 95% CI 1.41 to 2.69) and >2× ULN (HR 2.32, 95% CI 1.66 to 3.26). Relative to patients with M1a disease, patients with M1c non-CNS disease (HR 1.77, 95% CI 1.19–2.64) and M1c CNS disease (HR 1.54, 95% CI 0.97–2.45) had worse PFS. Similar trends in relation to OS were observed for pre-treatment metastatic site and extent of LDH elevation (see Fig. 2).

### Efficacy of post-HD IL-2 therapies

**Fig. 3:**
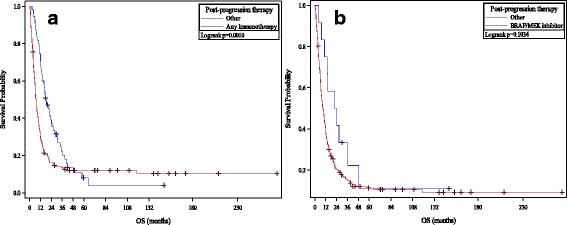
OS Analyses By Post HD IL-2 Therapy. **a** and **b** Kaplan-Meier plots of overall survival in patients who progress on HD IL-2 therapy depending on receipt of CTLA-4/PD-1 checkpoint inhibitor therapy (**a**) or BRAF/MEK inhibitors (**b**). CTLA-4/PD-1 checkpoint inhibitor therapy in HD IL-2 failures prolongs survival compared to untreated patients; with similar 1−/2−/3- year survival rates as those treated independently. BRAF/MEK inhibitor therapy in HD IL-2 failures produces similar PFS benefits but overall survival is not significantly improved

The extent of progression (systemic vs. CNS vs. systemic and CNS) was known in 189 (78%) patients. There was no significant difference between patients who progressed with CNS metastases and those who progressed systemically (*p* = 0.056 for OS, 0.97 for PFS). Information on post-progression therapies was available on 210 (86%) patients (see Fig. 3). Thirty-six patients were treated with CTLA-4 and/or PD-1 inhibitors while 12 patients were treated with BRAF or BRAF/MEK inhibitors. Of the 36 patients treated with CTLA-4 or PD-1 inhibitors, 7 (19%) patients remain alive with ongoing response to CTLA-4 or PD-1 blockade similar to previously published data regarding the clinical benefit of ipilimumab and pembrolizumab in HD IL-2 progressors [21]. The survival rates of patients who received CTLA-4/PD-1 checkpoint inhibitor therapy following progression on HD IL-2 (1−/2−/3- year survival rates of 78%/55%/32%) were similar to those published for these agents independently, suggesting that failure with HD IL-2 does not impede response to these agents [22–24]. Among the 12 patients with *BRAF* mutant melanoma who received BRAF/MEK inhibitors, median duration on therapy was 8.0 months – suggesting that these therapies retain their efficacy in patients who progress on HD IL-2.

### Incidence and efficacy of HD IL-2 in CNS metastatic melanoma

Data pertaining to the development and management of CNS metastases were available on 240 patients in whom he incidence of CNS metastases was 38% (see Additional file 1: Table S5). There was no substantial difference in PFS between patients who did (median PFS 3.0, 95% CI 2.1–3.7) and those who did not (median PFS 2.6, 95% CI 1.9–3.7) develop CNS metastases (*p* = 0.056). A small fraction of patients with treated CNS metastases had durable long-term PFS although no significant differences were noted between patients who developed CNS metastases prior to or after HD IL-2 therapy (*p* = 0.41).

## Discussion

To our knowledge, this collection of 243 melanoma patients treated with HD IL-2 is the largest institutional series to date to correlate response with PFS/OS that spanned the era before and after the introduction of effective therapies targeting driver mutations (BRAF/MEK inhibitors) and negative regulatory checkpoints (CTLA-4/PD-1 inhibitors). OS rates were compared between patients treated before and after these agents were available on clinical trials at the parent institution (2006) and following regulatory approval (2010). OS rates were unchanged in patients treated before 2006 and after 2007. However, compared to patients treated before 2010, patients treated after 2011 had improved OS (*p* = 0.0053), likely reflecting the increased availability of highly effective therapies such as CTLA-4/PD-1 and BRAF/MEK inhibitors in the community. Two other large series have studied the efficacy of HD IL-2 in patients with melanoma and renal cell carcinoma (RCC): Providence Cancer Center (314 melanoma patients) [25] and a more recent PROCLAIM^SM^ registry (170 melanoma patients) study [26]. Interestingly, our responses rates were similar to those in the more recently reported Alva et al. study, both of which were lower than those reported in the older Providence Cancer Center study; differences possibly explained by relative dose intensity as inferred from pressor use and patient selection factors inherent in the Providence Cancer Center study study that primarily comprised patients treated before the advent of modern therapies.

Consistent with prior reports from the Surgical Branch of the NCI and other large series, OS curves in this analysis show a clear plateau that began around 36 months following initiation of therapy [25–27]. Patients who reached this survival landmark - 14% in this analysis - appear to have a low-risk of subsequent relapse/death. Unfortunately, in the absence of ongoing surveillance imaging, it is impossible to clarify whether these patients are truly disease-free or have low-level residual tumor. Median OS (10.5 months vs. 8.0 months) and 3-year survival rates (15% and 13%) were similar for patients treated in the 1st or 2nd/subsequent lines. However post-hoc analyses do not account for potential differences of key prognostic factors between groups precluding definitive conclusions from being drawn regarding the relative benefits of using HD IL-2 in the 1st or subsequent line setting.

Presence of elevated LDH and/or visceral/CNS metastases prior to HD IL-2 were associated with poorer OS. Trend analyses suggested that the degree of LDH elevation appeared to have prognostic impact: patients with >2× ULN LDH values had significantly poorer PFS/OS than patients with 1-2× ULN LDH, who in turn did worse than patients with normal LDH. Similarly, patients with M1c (CNS and non-CNS) metastases had significantly poorer PFS/OS than patients with M1a and M1b disease. Although the presence of CNS disease was correlated with an adverse outcome, a subset of patients with treated CNS disease had durable long-term remissions consistent with prior reports of CNS activity of HD IL-2 [28, 29].

Various groups have evaluated utility of predictive biomarkers to predict benefit to HD IL-2 in RCC and melanoma. Several factors including disease burden, alveolar growth pattern and indolent disease are associated with increased rates of response to HD IL-2 in RCC although carbonic anhydrase IX appears to be more prognostic than predictive of HD IL-2 response. However, a prospective biomarker validation study (HD IL-2 “SELECT”) based on this “integrated selection” model (ISM) concluded that response rates did not significantly differ by ISM classification [30]. A similar classifier based on gene expression profiling in melanoma patients treated with HD IL-2 had previously reported greater response rates in tumors that expressed certain genes including Annexin A1, IL6R, oncostatin M, MCSF and GMCSF (class 2) compared to tumors that expressed MITF and melanocyte antigen expression (class 1) [31]; prospective validation of which is pending at this time. Given the small number of *NRAS* mutant patients in our series, we were not able to independently validate its favorable impact in HD IL-2 treated patients [20].

Fundamental advances in tumor immunology identified negative regulatory checkpoints (CTLA-4/PD-1) as key mediators of immune escape. Blocking antibodies to CTLA-4 (ipilimumab) and PD-1 (pembrolizumab and nivolumab) have reported durable survival rates of 20%–23% and 25%–30% at 3 years respectively [22–24]. Long term follow-up studies suggest that responses are durable especially if ongoing past 36–48 months – similar to those observed with HD IL-2 in this and other series. HD IL-2 is efficacious in a small proportion of patients who have progressed past either CTLA-4 or PD-1/PD-L1 checkpoint inhibitors [32] – an approach is being prospectively evaluated in a Cytokine Working Group study (NCT02796352).

In the current landscape, superior response rates observed with BRAF/MEK inhibitors and PD-1/CTLA-4 inhibitors have resulted in these agents supplanting HD IL-2 in the treatment of melanoma patients; particularly when coupled with the attendant complexities of HD IL-2 administration. However, identification of predictive biomarkers and/or established efficacy in a PD-1 refractory cohort may result in HD IL-2 remaining in the melanoma therapeutic armamentarium.

Separately, a modified form of aldesleukin has been developed in which conjugation with 6 releasable polyethylene glycol (PEG) chains results in a moiety (NKTR-214) that provides significant greater tumor exposure with less frequent dosing (relative to aldesleukin) with interesting in vitro activity singly and in combination with immune checkpoint blockade [33]. An early dose-finding study reported single-agent activity in RCC and melanoma with favorable safety and tolerability profile coupled with convenient outpatient administration schedule although this data has not been published [34].

In conclusion, this pooled analysis of patients with advanced melanoma treated with HD IL-2 in the modern era adds to the available data indicating durable long-term survival in responders. Median PFS/OS were 2.8 months and 9.6 months respectively. OS curves plateaued after 3 years - akin to the pattern observed in the pooled analyses of melanoma patients treated with CTLA-4 inhibitor ipilimumab and PD-1 inhibitors nivolumab and pembrolizumab, albeit at a lower fraction [22–24]. Degree of LDH elevation and M1c disease portend for poorer survival outcomes with HD IL-2 and should be considered in evaluating ongoing/future trials of this agent and possibly other immunotherapeutic agents including checkpoint inhibitors. Although numbers are small, the fraction of responders to PD-1/CTLA-4 blockade and duration of response to BRAF (or BRAF + MEK) inhibitors following progression on HD IL-2 is consistent with other reports.

## Conclusions

Immune checkpoint blockade has transformed the management of advanced melanoma with durable responses in up to 40% of treated patients. However, not all patients respond and no validated predictive biomarkers exist. HD IL-2 remains a viable treatment in melanoma and may be safely administered in a non-ICU setting. Pre-treatment disease burden and LDH elevation may select for patients at greater likelihood of benefit to HD IL-2.

## Additional file

Additional file 1: Table S1. (DLT Distribution of Patients Treated with HD IL-2); **Table S2.** (Overall Responses to HD IL-2 Therapy in Evaluable Patients By Baseline Characteristics (*N* = 237)); **Table S3.** (PFS Analyses By Baseline Characteristics); **Table S4.** (OS Analyses By Baseline Characteristics); and **Table S5.** (Incidence of CNS Metastases (*N* = 240)). (DOCX 57 kb)

## Abbreviations

AJCC: American Joint Committee on Cancer
CI: Confidence intervals
CNS: Central nervous system
CR: Complete response
CTLA-4: Cytotoxic T-lymphocyte antigen 4
DLT: Dose-limiting toxicities
HD IL-2: High-dose interleukin-2
ICU: Intensive care unit
IRB: Institutional Review Board
LDH: Lactate dehydrogenase
OR: Overall response
OS: Overall survival
PD-1: Programmed death 1
PFS: Progression-free survival
RECIST: Response Evaluation Criteria in Solid Tumors
SOC: Standard-of-care
ULN: Upper limit normal
UPCI: University of Pittsburgh Cancer Institute

## References

[1] Lotze MT, Frana LW, Sharrow SO, Robb RJ, Rosenberg SA (1985). In vivo administration of purified human interleukin 2. I. Half-life and immunologic effects of the Jurkat cell line-derived interleukin 2. J Immunol.

[2] Lotze MT, Matory YL, Ettinghausen SE, Rayner AA, Sharrow SO, Seipp CA, Custer MC, Rosenberg SA (1985). In vivo administration of purified human interleukin 2. II. Half life, immunologic effects, and expansion of peripheral lymphoid cells in vivo with recombinant IL 2. J Immunol.

[3] Atkins MB, Lotze MT, Dutcher JP, Fisher RI, Weiss G, Margolin K, Abrams J, Sznol M, Parkinson D, Hawkins M, Paradise C, Kunkel L, Rosenberg SA (1999). High-dose recombinant interleukin 2 therapy for patients with metastatic melanoma: analysis of 270 patients treated between 1985 and 1993. J Clin Oncol.

[4] Rosenberg SA, Yang JC, Topalian SL, Schwartzentruber DJ, Weber JS, Parkinson DR, Seipp CA, Einhorn JH, White DE (1994). Treatment of 283 consecutive patients with metastatic melanoma or renal cell cancer using high-dose bolus interleukin 2. JAMA.

[5] Chapman PB, Hauschild A, Robert C, Haanen JB, Ascierto P, Larkin J, Dummer R, Garbe C, Testori A, Maio M, Hogg D, Lorigan P, Lebbe C, Jouary T, Schadendorf D, Ribas A, O’Day SJ, Sosman JA, Kirkwood JM, Eggermont AM, Dreno B, Nolop K, Li J, Nelson B, Hou J, Lee RJ, Flaherty KT, McArthur GA (2011). BRIM-3 study group: improved survival with vemurafenib in melanoma with BRAF V600E mutation. N Engl J Med.

[6] Flaherty KT, Robert C, Hersey P, Nathan P, Garbe C, Milhem M, Demidov LV, Hassel JC, Rutkowski P, Mohr P, Dummer R, Trefzer U, Larkin JM, Utikal J, Dreno B, Nyakas M, Middleton MR, Becker JC, Casey M, Sherman LJ, Wu FS, Ouellet D, Martin AM, Patel K, Schadendorf D (2012). METRIC study group: improved survival with MEK inhibition in BRAF-mutated melanoma. N Engl J Med.

[7] Flaherty KT, Infante JR, Daud A, Gonzalez R, Kefford RF, Sosman J, Hamid O, Schuchter L, Cebon J, Ibrahim N, Kudchadkar R, Burris HA, Falchook G, Algazi A, Lewis K, Long GV, Puzanov I, Lebowitz P, Singh A, Little S, Sun P, Allred A, Ouellet D, Kim KB, Patel K, Weber J (2012). Combined BRAF and MEK inhibition in melanoma with BRAF V600 mutations. N Engl J Med.

[8] Long GV, Stroyakovskiy D, Gogas H, Levchenko E, de Braud F, Larkin J, Garbe C, Jouary T, Hauschild A, Grob JJ, Chiarion Sileni V, Lebbe C, Mandalà M, Millward M, Arance A, Bondarenko I, Haanen JB, Hansson J, Utikal J, Ferraresi V, Kovalenko N, Mohr P, Probachai V, Schadendorf D, Nathan P, Robert C, Ribas A, DeMarini DJ, Irani JG, Casey M (2014). Combined BRAF and MEK inhibition versus BRAF inhibition alone in melanoma. N Engl J Med.

[9] Robert C, Karaszewska B, Schachter J, Rutkowski P, Mackiewicz A, Stroiakovski D, Lichinitser M, Dummer R, Grange F, Mortier L, Chiarion-Sileni V, Drucis K, Krajsova I, Hauschild A, Lorigan P, Wolter P, Long GV, Flaherty K, Nathan P, Ribas A, Martin AM, Sun P, Crist W, Legos J, Rubin SD, Little SM, Schadendorf D (2015). Improved overall survival in melanoma with combined dabrafenib and trametinib. N Engl J Med.

[10] Long GV, Stroyakovskiy D, Gogas H, Levchenko E, de Braud F, Larkin J, Garbe C, Jouary T, Hauschild A, Grob JJ, Chiarion-Sileni V, Lebbe C, Mandalà M, Millward M, Arance A, Bondarenko I, Haanen JB, Hansson J, Utikal J, Ferraresi V, Kovalenko N, Mohr P, Probachai V, Schadendorf D, Nathan P, Robert C, Ribas A, DJ DM, Irani JG, Swann S (2015). Dabrafenib and trametinib versus dabrafenib and placebo for Val600 BRAF-mutant melanoma: a multicentre, double-blind, phase 3 randomised controlled trial. Lancet.

[11] Hodi FS, O’Day SJ, McDermott DF, Weber RW, Sosman JA, Haanen JB, Gonzalez R, Robert C, Schadendorf D, Hassel JC, Akerley W, van den Eertwegh AJ, Lutzky J, Lorigan P, Vaubel JM, Linette GP, Hogg D, Ottensmeier CH, Lebbé C, Peschel C, Quirt I, Clark JI, Wolchok JD, Weber JS, Tian J, Yellin MJ, Nichol GM, Hoos A, Urba WJ (2010). Improved survival with ipilimumab in patients with metastatic melanoma. N Engl J Med.

[12] Ribas A, Puzanov I, Dummer R, Schadendorf D, Hamid O, Robert C, Hodi FS, Schachter J, Pavlick AC, Lewis KD, Cranmer LD, Blank CU, O’Day SJ, Ascierto PA, Salama AK, Margolin KA, Loquai C, Eigentler TK, Gangadhar TC, Carlino MS, Agarwala SS, Moschos SJ, Sosman JA, Goldinger SM, Shapira-Frommer R, Gonzalez R, Kirkwood JM, Wolchok JD, Eggermont A, Li XN (2015). Pembrolizumab versus investigator-choice chemotherapy for ipilimumab-refractory melanoma (KEYNOTE-002): a randomised, controlled, phase 2 trial. Lancet Oncol.

[13] Weber JS, SP D’ A, Minor D, Hodi FS, Gutzmer R, Neyns B, Hoeller C, Khushalani NI, Miller WH, Lao CD, Linette GP, Thomas L, Lorigan P, Grossmann KF, Hassel JC, Maio M, Sznol M, Ascierto PA, Mohr P, Chmielowski B, Bryce A, Svane IM, Grob JJ, Krackhardt AM, Horak C, Lambert A, Yang AS, Larkin J (2015). Nivolumab versus chemotherapy in patients with advanced melanoma who progressed after anti-CTLA-4 treatment (CheckMate 037): a randomised, controlled, open-label, phase 3 trial. Lancet Oncol.

[14] Larkin J, Chiarion-Sileni V, Gonzalez R, Grob JJ, Cowey CL, Lao CD, Schadendorf D, Dummer R, Smylie M, Rutkowski P, Ferrucci PF, Hill A, Wagstaff J, Carlino MS, Haanen JB, Maio M, Marquez-Rodas I, McArthur GA, Ascierto PA, Long GV, Callahan MK, Postow MA, Grossmann K, Sznol M, Dreno B, Bastholt L, Yang A, Rollin LM, Horak C, Hodi FS (2015). Combined Nivolumab and Ipilimumab or Monotherapy in untreated melanoma. N Engl J Med.

[15] Tarhini AA, Kirkwood JM, Gooding WE, Cai C, Agarwala SS (2007). Durable complete responses with high-dose bolus interleukin-2 in patients with metastatic melanoma who have experienced progression after biochemotherapy. J Clin Oncol.

[16] Tarhini AA, Kirkwood JM, Gooding WE, Moschos S, Agarwala SS (2008). A phase 2 trial of sequential temozolomide chemotherapy followed by high-dose interleukin 2 immunotherapy for metastatic melanoma. Cancer.

[17] Schwartzentruber DJ (2001). Guidelines for the safe administration of high-dose interleukin-2. J Immunother.

[18] Balch CM, Gershenwald JE, Soong S-J, Thompson JF, Atkins MB, Byrd DR, Buzaid AC, Cochran AJ, Coit DG, Ding S, Eggermont AM, Flaherty KT, Gimotty PA, Kirkwood JM, McMasters KM, Mihm MC, Morton DL, Ross MI, Sober AJ, Sondak VK. Final version of 2009 AJCC melanoma staging and classification. J Clin Oncol. 2009(27):6199–206. 10.1200/JCO.2009.23.4799 PMC2793035 19917835

[19] Eisenhauer EA, Therasse P, Bogaerts J, Schwartz LH, Sargent D, Ford R, Dancey J, Arbuck S, Gwyther S, Mooney M, Rubinstein L, Shankar L, Dodd L, Kaplan R, Lacombe D, Verweij J (2009). New response evaluation criteria in solid tumours: revised RECIST guideline (version 1.1). Eur J Cancer.

[20] Joseph RW, Sullivan RJ, Harrell R, Stemke-Hale K, Panka D, Manoukian G, Percy A, Bassett RL, Ng CS, Radvanyi L, Hwu P, Atkins MB, Davies MA (2012). Correlation of NRAS mutations with clinical response to high-dose IL-2 in patients with advanced melanoma. J Immunother.

[21] Joseph RW, Eckel-Passow JE, Sharma R, Liu P, Parker A, Jakob J, Buchbinder E, Bassett RL, Davies MA, Hwu P, Atkins MB, Sullivan RJ (2012). Characterizing the clinical benefit of ipilimumab in patients who progressed on high-dose IL-2. J Immunother.

[22] Schadendorf D, Hodi FS, Robert C, Weber JS, Margolin K, Hamid O, Patt D, Chen T-T, Berman DM, Wolchok JD (2015). Pooled analysis of long-term survival data from phase II and phase III trials of Ipilimumab in Unresectable or metastatic melanoma. J Clin Oncol.

[23] Ribas A, Hamid O, Daud A, Hodi FS, Wolchok JD, Kefford R, Joshua AM, Patnaik A, Hwu W-J, Weber JS, Gangadhar TC, Hersey P, Dronca R, Joseph RW, Zarour H, Chmielowski B, Lawrence DP, Algazi A, Rizvi NA, Hoffner B, Mateus C, Gergich K, Lindia JA, Giannotti M, Li XN, Ebbinghaus S, Kang SP, Robert C (2016). Association of pembrolizumab with tumor response and survival among patients with advanced melanoma. JAMA.

[24] Larkin J, Lao CD, Urba WJ, McDermott DF, Horak C, Jiang J, Wolchok JD (2015). Efficacy and safety of Nivolumab in patients with BRAF V600 mutant and BRAF wild-type advanced melanoma: a pooled analysis of 4 clinical trials. JAMA Oncol.

[25] Payne R, Glenn L, Hoen H, Richards B, Smith JW, Lufkin R, Crocenzi TS, Urba WJ, Curti BD (2014). Durable responses and reversible toxicity of high-dose interleukin-2 treatment of melanoma and renal cancer in a community hospital biotherapy program. J Immunother Cancer.

[26] Alva A, Daniels GA, Wong MKK, Kaufman HL, Morse MA, McDermott DF, Clark JI, Agarwala SS, Miletello G, Logan TF, Hauke RJ, Curti B, Kirkwood JM, Gonzalez R, Amin A, Fishman M, Agarwal N, Lowder JN, Hua H, Aung S, Dutcher JP (2016). Contemporary experience with high-dose interleukin-2 therapy and impact on survival in patients with metastatic melanoma and metastatic renal cell carcinoma. Cancer Immunol Immunother.

[27] Rosenberg SA (2012). Raising the bar: the curative potential of human cancer immunotherapy. Sci Transl Med.

[28] Powell S, Dudek AZ (2009). Single-institution outcome of high-dose interleukin-2 (HD IL-2) therapy for metastatic melanoma and analysis of favorable response in brain metastases. Anticancer Res.

[29] Chu MB, Fesler MJ, Armbrecht ES, Fosko SW, Hsueh E, Richart JM (2013). High-dose interleukin-2 (HD IL-2) therapy should be considered for treatment of patients with melanoma brain metastases. Chemother Res Pract.

[30] DF MD, Cheng SC, Signoretti S, Margolin KA, Clark JI, Sosman JA, Dutcher JP, Logan TF, Curti BD, Ernstoff MS, Appleman L, Wong MK, Khushalani NI, Oleksowicz L, Vaishampayan UN, Mier JW, Panka DJ, Bhatt RS, Bailey AS, Leibovich BC, Kwon ED, Kabbinavar FF, Belldegrun AS, Figlin RA, Pantuck AJ, Regan MM, Atkins MB (2015). The high-dose aldesleukin “select” trial: a trial to prospectively validate predictive models of response to treatment in patients with metastatic renal cell carcinoma. Clin Cancer Res.

[31] Sullivan RJ, Hoshida Y, Brunet J, Tahan S, Alridge J, Kwabi C, Gardiner E, McDermott D, Golub T, Atkins MB. A single center experience with high-dose (HD) IL-2 treatment for patients with advanced melanoma and pilot investigation of a novel gene expression signature as a predictor of response. Presented at: 2009 ASCO Annual Meeting; May 2009. Abstract 9003.

[32] Buchbinder EI, Gunturi A, Perritt J, Dutcher J, Aung S, Kaufman HL, Ernstoff MS, Miletello GP, Curti BD, Daniels GA, Patel SP, Kirkwood JM, Hallmeyer S, Clark JI, Gonzalez R, Richart JM, Lutzky J, Morse MA, Sullivan RJ, McDermott DF (2016). A retrospective analysis of high-dose interleukin-2 (HD IL-2) following Ipilimumab in metastatic melanoma. J Immunother Cancer.

[33] Charych DH, Hoch U, Langowski JL, Lee SR, Addepalli MK, Kirk PB, Sheng D, Liu X, Sims PW, VanderVeen LA, Ali CF, Chang TK, Konakova M, Pena RL, Kanhere RS, Kirksey YM, Ji C, Wang Y, Huang J, Sweeney TD, Kantak SS, Doberstein SK (2016). NKTR-214, an engineered cytokine with biased IL2 receptor binding, increased tumor exposure, and marked efficacy in mouse tumor models. Clin Cancer Res.

[34] Bernatchez C, Haymaker C, Tannir NM, et al. A CD122-biased agonist increases CD8+T Cells and natural killer cells in the tumor microenvironment; making cold tumors hot with NKTR-214. Presented at: 2016 SITC Annual Meeting; November 9–13, 2016; National Harbor, MD. Abstract 387.

