# Supplementary material for: High-dose interleukin-2 (HD IL-2) for advanced melanoma: a single center experience from the University of Pittsburgh Cancer Institute
Source: J Immunother Cancer. 2017 Sep 19;5:74. doi: 10.1186/s40425-017-0279-5 (PMC5604296; doi:10.1186/s40425-017-0279-5)
Supplement: Additional file 1: Table S1. — (DLT Distribution of Patients Treated with HD IL-2); Table S2. (Overall Responses to HD IL-2 Therapy in Evaluable Patients By Baseline Characteristics (N = 237)); Table S3. (PFS Analyses By Baseline Characteristics); Table S4. (OS Analyses By Baseline Characteristics); and Table S5. (Incidence of CNS Metastases (N = 240)). (DOCX 57 kb) [file 40425_2017_279_MOESM1_ESM.docx]

| **Supplementary Table 1: DLT Distribution of Patients Treated with HD IL-2** | | | | | | | | | | | | | | | | | | |
| --- | --- | --- | --- | --- | --- | --- | --- | --- | --- | --- | --- | --- | --- | --- | --- | --- | --- | --- |
| **DLT distribution** | **Cycle 1** | **% (/pts in each cycle)** | **% (/doses in cycle)** | **Cycle 2** | **% (/pts in each cycle)** | **% (/doses in cycle)** | **Cycle 3** | **% (/pts in each cycle)** | **% (/doses in cycle)** | **Cycle 4** | **% (/pts in each cycle)** | **% (/doses in cycle)** | **Cycle 5** | **% (/pts in each cycle)** | **% (/doses in cycle)** | **Cycle 6** | **% (/pts in each cycle)** | **% (/doses in cycle)** |
| **1. CNS** |  |  |  |  |  |  |  |  |  |  |  |  |  |  |  |  |  |  |
| **1.1 confusion/delirium** | 12 | 4.9 | 0.6 | 4 | 2.0 | 0.3 | 2 | 2.1 | 0.3 | 4 | 4.6 | 0.7 | 1 | 2.6 | 0.4 | 0 | 0.0 | 0.0 |
| **1.2 hallucinations** | 5 | 2.1 | 0.2 | 6 | 3.0 | 0.4 | 4 | 4.1 | 0.5 | 3 | 3.4 | 0.5 | 3 | 7.7 | 1.1 | 1 | 3.2 | 0.5 |
| **1.3 seizures** | 0 | 0.0 | 0.0 | 0 | 0.0 | 0.0 | 0 | 0.0 | 0.0 | 0 | 0.0 | 0.0 | 0 | 0.0 | 0.0 | 0 | 0.0 | 0.0 |
| **2. CVS** |  |  |  |  |  |  |  |  |  |  |  |  |  |  |  |  |  |  |
| **1.1 arrhythmia** | 6 | 2.5 | 0.3 | 5 | 2.5 | 0.3 | 3 | 3.1 | 0.4 | 3 | 3.4 | 0.5 | 3 | 7.7 | 1.1 | 1 | 3.2 | 0.5 |
| **1.2 tachycardia** | 43 | 17.7 | 2.1 | 42 | 20.7 | 2.9 | 19 | 19.6 | 2.6 | 17 | 19.5 | 3.0 | 4 | 10.3 | 1.5 | 3 | 9.7 | 1.5 |
| **1.2 hypotension** | 33 | 13.6 | 1.6 | 42 | 20.7 | 2.9 | 22 | 22.7 | 3.0 | 27 | 31.0 | 4.8 | 10 | 25.6 | 3.6 | 12 | 38.7 | 5.9 |
| **1.3 pulmonary edema** | 8 | 3.3 | 0.4 | 2 | 1.0 | 0.1 | 5 | 5.2 | 0.7 | 0 | 0.0 | 0.0 | 3 | 7.7 | 1.1 | 1 | 3.2 | 0.5 |
| **1.4 weight gain (>15%)** | 46 | 18.9 | 2.2 | 43 | 21.2 | 3.0 | 12 | 12.4 | 1.6 | 8 | 9.2 | 1.4 | 4 | 10.3 | 1.5 | 2 | 6.5 | 1.0 |
| **1.5 edema** | 37 | 15.2 | 1.8 | 23 | 11.3 | 1.6 | 16 | 16.5 | 2.2 | 8 | 9.2 | 1.4 | 5 | 12.8 | 1.8 | 3 | 9.7 | 1.5 |
| **3. RESPIRATORY** |  |  |  |  |  |  |  |  |  |  |  |  |  |  |  |  |  |  |
| **3.1 SOB (subjective)** | 27 | 11.1 | 1.3 | 15 | 7.4 | 1.0 | 3 | 3.1 | 0.4 | 2 | 2.3 | 0.4 | 1 | 2.6 | 0.4 | 1 | 3.2 | 0.5 |
| **3.2 ARDS** | 2 | 0.8 | 0.1 | 0 | 0.0 | 0.0 | 0 | 0.0 | 0.0 | 0 | 0.0 | 0.0 | 0 | 0.0 | 0.0 | 0 | 0.0 | 0.0 |
| **4. HEME** |  |  |  |  |  |  |  |  |  |  |  |  |  |  |  |  |  |  |
| **4.1 anemia** | 1 | 0.4 | 0.0 | 0 | 0.0 | 0.0 | 0 | 0.0 | 0.0 | 1 | 1.1 | 0.2 | 0 | 0.0 | 0.0 | 0 | 0.0 | 0.0 |
| **4.2 neutropenia** | 3 | 1.2 | 0.1 | 1 | 0.5 | 0.1 | 0 | 0.0 | 0.0 | 0 | 0.0 | 0.0 | 0 | 0.0 | 0.0 | 0 | 0.0 | 0.0 |
| **4.3 thrombocytopenia** | 27 | 11.1 | 1.3 | 7 | 3.4 | 0.5 | 6 | 6.2 | 0.8 | 3 | 3.4 | 0.5 | 2 | 5.1 | 0.7 | 0 | 0.0 | 0.0 |
| **5. GI** |  |  |  |  |  |  |  |  |  |  |  |  |  |  |  |  |  |  |
| **5.1 nausea/vomiting** | 9 | 3.7 | 0.4 | 8 | 3.9 | 0.6 | 6 | 6.2 | 0.8 | 3 | 3.4 | 0.5 | 2 | 5.1 | 0.7 | 2 | 6.5 | 1.0 |
| **5.2 diarrhea** | 15 | 6.2 | 0.7 | 14 | 6.9 | 1.0 | 8 | 8.2 | 1.1 | 1 | 1.1 | 0.2 | 2 | 5.1 | 0.7 | 1 | 3.2 | 0.5 |
| **5.3 hyperbilirubinemia** | 9 | 3.7 | 0.4 | 7 | 3.4 | 0.5 | 3 | 3.1 | 0.4 | 1 | 1.1 | 0.2 | 0 | 0.0 | 0.0 | 0 | 0.0 | 0.0 |
| **5.4 transaminitis** | 2 | 0.8 | 0.1 | 0 | 0.0 | 0.0 | 0 | 0.0 | 0.0 | 0 | 0.0 | 0.0 | 1 | 2.6 | 0.4 | 0 | 0.0 | 0.0 |
| **6. RENAL** |  |  |  |  |  |  |  |  |  |  |  |  |  |  |  |  |  |  |
| **6.1 creatinine elevation** | 3 | 1.2 | 0.1 | 5 | 2.5 | 0.3 | 4 | 4.1 | 0.5 | 1 | 1.1 | 0.2 | 1 | 2.6 | 0.4 | 1 | 3.2 | 0.5 |
| **6.2 oliguria** | 35 | 14.4 | 1.7 | 61 | 30.0 | 4.2 | 0 | 0.0 | 0.0 | 34 | 39.1 | 6.1 | 15 | 38.5 | 5.5 | 18 | 58.1 | 8.9 |
| **6.3 hypocalcemia** | 1 | 0.4 | 0.0 | 0 | 0.0 | 0.0 | 0 | 0.0 | 0.0 | 0 | 0.0 | 0.0 | 0 | 0.0 | 0.0 | 0 | 0.0 | 0.0 |
| **6.4 hypomagnesemia** | 1 | 0.4 | 0.0 | 0 | 0.0 | 0.0 | 0 | 0.0 | 0.0 | 0 | 0.0 | 0.0 | 0 | 0.0 | 0.0 | 0 | 0.0 | 0.0 |
| **6.5 hypophosphatemia** | 1 | 0.4 | 0.0 | 0 | 0.0 | 0.0 | 0 | 0.0 | 0.0 | 0 | 0.0 | 0.0 | 0 | 0.0 | 0.0 | 0 | 0.0 | 0.0 |
| **6.6 metabolic acidosis** | 1 | 0.4 | 0.0 | 1 | 0.4 | 0.1 | 1 | 1.0 | 0.1 | 0 | 0.0 | 0.0 | 0 | 0.0 | 0.0 | 0 | 0.0 | 0.0 |
| **7.0 CONSTITUTIONAL** |  |  |  |  |  |  |  |  |  |  |  |  |  |  |  |  |  |  |
| **7.1 chills/rigors** | 4 | 1.6 | 0.2 | 5 | 2.5 | 0.3 | 3 | 3.1 | 0.4 | 0 | 0.0 | 0.0 | 1 | 2.6 | 0.4 | 1 | 3.2 | 0.5 |
| **7.2 fatigue** | 29 | 11.9 | 1.4 | 33 | 16.3 | 2.3 | 14 | 14.4 | 1.9 | 19 | 21.8 | 3.4 | 4 | 10.3 | 1.5 | 2 | 6.5 | 1.0 |
| **7.3 rash** | 6 | 2.5 | 0.3 | 2 | 1.0 | 0.1 | 1 | 1.0 | 0.1 | 0 | 0.0 | 0.0 | 0 | 0.0 | 0.0 | 0 | 0.0 | 0.0 |
| **7.4 fever** | 4 | 1.6 | 0.2 | 5 | 2.5 | 0.3 | 1 | 1.0 | 0.1 | 2 | 2.3 | 0.4 | 1 | 2.6 | 0.4 | 1 | 3.2 | 0.5 |
| **N (total number of patients receiving doses, per cycle)** | **243** |  |  | **203** |  |  | **97** |  |  | **87** |  |  | **39** |  |  | **31** |  |  |
| **N (total number of doses received, per cycle)** | **2087** |  |  | **1450** |  |  | **729** |  |  | **558** |  |  | **274** |  |  | **202** |  |  |
| **N (total number of cycles delivered): 5300** | | | | | | | | | | | | | | | | | | |

| **Supplementary Table 2: Overall Responses to HD IL-2 Therapy in Evaluable Patients By Baseline Characteristics (N=237)** | | | | |
| --- | --- | --- | --- | --- |
| *Histology* | | | | |
| BORR – number (%)   - CR - PR - SD - PD   No. CR/PR (%, 95% CI)  No. CR/PR/SD (%, 95% CI) | *Cutaneous (N=182)*  17 (9)  18 (10)  37 (20)  110 (60)  35 (19, 95% CI 14-26)  72 (40, 95% CI 32-47) | *Mucosal (N=14)*  1 (7)  2 (14)  4 (29)  7 (50)  3 (21, 95% CI 5-51)  7 (50, 95% CI 23-77) | *Uveal (N=16)*  0 (0)  1 (6)  5 (31)  10 (63)  1 (6, 95% CI 0-30)  6 (38, 95% CI 15-65) | *Unknown (N=24)*  1 (4)  3 (13)  8 (33)  12 (50)  4 (17, 95% CI 5-37)  12 (50, 95% CI 29-71) |
| *Line of Therapy* | | | | |
| BORR – number (%)   - CR - PR - SD - PD   No. CR/PR (%, 95% CI)  No. CR/PR/SD (%, 95% CI) | *1^st^ line (N=111)*  10 (9)  15 (14)  26 (23)  60 (54)  25 (23, 95% CI 15-31)  51 (46, 95% CI 36-56) | *2^nd^ line (N=80)*  3 (4)  6 (8)  18 (23)  53 (66)  9 (11, 95% CI 5-20)  27 (34, 95% CI 24-45) | *2nd or greater line (N=126)*  9 (7)  9 (7)  28 (22)  80 (63)  18 (14, 95% CI 9-22)  46 (37, 95% CI 28-46) | *3rd or greater line (N=46)*  6 (13)  3 (7)  10 (22)  27 (59)  9 (20, 95% 9-34)  19 (41, 95% CI 27-57) |
| *BRAF/NRAS mutation status* | | | | |
| BORR – number (%)   - CR - PR - SD - PD   No. CR/PR (%, 95% CI)  No. CR/PR/SD (%, 95% CI) | *BRAF mutant (N=29)*  3 (10)  6 (21)  5 (17)  15 (52)  9 (31, 95% CI 15-51)  14 (48, 95% CI 29-67) | *BRAF WT (N=22)*  0 (0)  3 (14)  7 (32)  12 (55)  3 (14, 95% CI 3-35)  10 (45, 95% CI 24-68) | *NRAS mutant (N=1)*  0 (0)  0 (0)  0 (0)  1 (100)  0 (0, -)  0 (0, -) | *NRAS WT (N=36)*  3 (8)  7 (19)  7 (19)  19 (53)  10 (28, 95% CI 14-45)  17 (47, 95% CI 30-65) |

**Supplementary Table 3: PFS Analyses By Baseline Characteristics**For PFS analysis, 6 patients not evaluable for response/progression were excluded.

| **Population** | **Subject** | **Event** | **Median PFS**  **with 95% CI** | **1-yr Survival**  **with 95% CI** | **2-yr Survival**  **with 95% CI** | **3-yr Survival**  **with 95% CI** | **Hazard Ratio**  **With 95% CI** |
| --- | --- | --- | --- | --- | --- | --- | --- |
| Entire cohort | 237 | 213 | 2.8 (2.2-3.5) | 15% (11%-20%) | 10% (7%-14%) | 8% (5%-12%) | - |
| LDH |  |  |  |  |  |  |  |
| Normal | 104 | 86 | 5.2 (3.7-6.9) | 24% (16%-33%) | 17% (10%-25%) | 15% (8%-22%) | 1 |
| 1xULN-2xULN | 70 | 68 | 2.0 (1.6-3.0) | 9% (4%-17%) | 4% (1%-11%) | 1% (0.1%-7%) | 1.95 (1.41-2.69) |
| >2xULN | 63 | 59 | 1.7 (1.3-2.3) | 7% (2%-15%) | 5% (1%-13%) | 5% (1%-13%) | 2.32 (1.66-3.26) |
| Metastatic Status |  |  |  |  |  |  |  |
| Skin, subcutaneous, LN (M1a) | 42 | 34 | 3.4 (1.9-8.2) | 32% (18%-46%) | 26% (14%-41%) | 21% (10%-35%) | 1 |
| Lung (M1b) | 56 | 49 | 3.1 (2.0-6.0) | 17% (8%-28%) | 13% (6%-24%) | 9% (3%-19%) | 1.20 (0.77-1.86) |
| Non-lung visceral (M1c non-CNS) | 98 | 91 | 2.1 (1.6-2.9) | 11% (5%-18%) | 5% (2%-11%) | 5% (2%-11%) | 1.77 (1.19-2.64) |
| CNS (M1c CNS) | 41 | 39 | 3.7 (1.7-4.4) | 8% (2%-18%) | 3% (0.2% -11%) | 3% (0.2% -11%) | 1.54 (0.97-2.45) |
| CNS met# |  |  |  |  |  |  |  |
| No CNS met | 146 | 126 | 2.6 (2.1-3.7) | 20% (14%-28%) | 14% (8%-20%) | 12% (7%-18%) | 1 |
| CNS met prior to treatment | 41 | 39 | 3.7 (1.7-4.4) | 8% (2%-18%) | 3% (0.2%-11%) | 3% (0.2%-11%) | 1.21 (0.84-1.73) |
| CNS met after treatment | 49 | 48 | 2.1 (1.7-3.3) | 6% (2%-15%) | 6% (2%-15%) | 2% (0.2%-9%) | 1.41 (1.01-1.97) |
| Progression extent * |  |  |  |  |  |  |  |
| Systematic | 151 | 151 | 2.0 (1.7-2.6) | 8% (4%-13%) | 3% (1%-7%) | 0.7% (0.1%-3%) | 1 |
| CNS ** | 38 | 38 | 2.6 (1.8-4.2) | 3% (0.2%-12%) | 0% | 0% | 1.00 (0.70-1.44) |
| Unknown | 29 | 22 | 3.7 (2.4-7.5) | 21% (7%-39%) | 0% | 0% | 0.65 (0.42-1.02) |

#Patients in the unknown category were excluded

*patient in the N/A categories were excluded

**This category include CNS only progression and systemic and CNS progression

**Supplementary Table 4: OS Analyses By Baseline Characteristics**

**Median follow-up for entire cohort**: 9.4 months (range 0.2-273 months).

| **Population** | **Subject** | **Event** | **Median OS**  **with 95% CI** | **1-yr Survival**  **with 95% CI** | **2-yr Survival**  **with 95% CI** | **3-yr Survival**  **with 95% CI** | **Hazard Ratio**  **With 95% CI** |
| --- | --- | --- | --- | --- | --- | --- | --- |
| Entire cohort | 243 | 215 | 9.6 (7.4-11.2) | 41% (34%-47%) | 20% (15%-25%) | 14% (10%-19%) | -- |
| LDH |  |  |  |  |  |  |  |
| Normal | 106 | 88 | 13.6 (11.6-18.4) | 57% (47%-65%) | 30% (21%-39%) | 20% (13%-29%) | 1 |
| 1xULN-2xULN | 74 | 69 | 7.5 (6.1-10.6) | 37% (26%-48%) | 16% (9%-26%) | 12% (5%-20%) | 1.67 (1.22-2.29) |
| >2xULN | 63 | 58 | 4.9 (3.2-5.9) | 18% (10%-28%) | 8% (3%-17%) | 7% (2%-15%) | 2.49 (1.78-3.48) |
| Metastatic Status |  |  |  |  |  |  |  |
| Skin, subcutaneous, LN (M1a) | 43 | 33 | 24.2 (12.0-31.0) | 65% (49%-77%) | 51% (35%-65%) | 28% (15%-42%) | 1 |
| Lung (M1b) | 58 | 50 | 12.8 (10.5-16.7) | 53% (40%-65%) | 23% (13%-35%) | 17% (9%-28%) | 1.42 (0.91-2.21) |
| Non-lung visceral (M1c non-CNS) | 99 | 92 | 6.1 (4.9-7.8) | 30% (21%-39%) | 9% (4%-15%) | 9% (4%-15%) | 2.49 (1.67-3.73) |
| CNS (M1c CNS) | 43 | 40 | 6.8 (4.4-9.4) | 24% (12%-37%) | 10% (3%-21%) | 7% (2%-17%) | 2.62 (1.64-4.18) |
| CNS met % |  |  |  |  |  |  |  |
| No CNS | 148 | 124 | 10.2 (7.2-12.4) | 42% (34%-50%) | 24% (18%-31%) | 20% (13%-26%) | 1 |
| CNS met prior to treatment | 43 | 40 | 6.8 (4.4-9.4) | 24% (12%-37%) | 10% (3%-21%) | 7% (2%-17%) | 1.60 (1.12-2.29) |
| CNS met after treatment | 49 | 48 | 11.2 (7.8-14.4) | 49% (34%-62%) | 17% (8%-28%) | 6% (2%-15%) | 1.13 (0.81-1.58) |
| Progression extent * |  |  |  |  |  |  |  |
| Systematic | 151 | 141 | 10.2 (7.5-12.0) | 41% (33%-49%) | 18% (12%-24%) | 12% (7%-18%) | 1 |
| CNS ** | 38 | 38 | 7.3 (6.2-10.6) | 26% (14%-41%) | 11% (3%-23%) | 0% | 1.42 (0.99-2.04) |
| Unknown | 35 | 34 | 5.7 (2.7-7.5) | 26% (13%-42%) | 3% (0.2%-13%) | 0% | 1.98 (1.35-2.90) |
| Post-progression therapy# |  |  |  |  |  |  |  |
| Checkpoint immunotherapy | 36 | 29 | 24.7 (15.9-35.1) | 78% (60%-88%) | 55% (37%-70%) | 32% (17%-48%) | 1 |
| Other | 174 | 155 | 7.5 (6.4-9.9) | 33% (26%-40%) | 15% (10%-21%) | 12% (8%-18%) | 2.02 (1.35-3.01) |
| Any immunotherapy | 55 | 48 | 18.4 (13.2-24.7) | 71% (57%-81%) | 39% (26%-52%) | 22% (12%-34%) | 1 |
| Other | 155 | 136 | 7.2 (6.0-8.4) | 30% (23%-37%) | 16% (11%-22%) | 13% (9%-19%) | 1.73 (1.24-2.42) |
| BRAF/MEK inhibitor | 12 | 10 | 23.2 (7.4-36.2) | 75% (41%-91%) | 50% (21%-74%) | 33% (10%-59%) | 1 |
| Other | 198 | 174 | 9.0 (7.2-10.7) | 39% (32%-45%) | 20% (15%-26%) | 15% (10%-20%) | 1.69 (0.89-3.20) |
| Any targeted therapy | 16 | 14 | 18.0 (7.4-26.7) | 69% (40%-86%) | 38% (15%-60%) | 25% (8%-47%) | 1 |
| Other | 194 | 170 | 9.0 (7.2-10.7) | 38% (32%-45%) | 21% (15%-27%) | 15% (10%-21%) | 1.44 (0.83-2.48) |

%Patients in whom nature of progression was unknown excluded.

*Patients who had not progressed were excluded.

**This category include CNS only progression and systemic and CNS progression.

#Patients who had unknown post-progression therapy were excluded.

| **Supplementary Table 5: Incidence of CNS Metastases (N=240)** |
| --- |
| No CNS metastases* 148 (62%)  CNS metastases present 92 (38%)   - CNS metastases prior to HD IL-2 therapy 43 (18%) - CNS metastases during/after HD IL-2 therapy 49 (20.4%) |
| *Considered when ≥2 CNS imaging studies (MRI and/or CT) with at least 1 prior to HD IL-2 therapy and 1 during/after HD IL-2 therapy |
